# Simultaneous estimation of diet composition and calibration coefficients with fatty acid signature data

**DOI:** 10.1002/ece3.3179

**Published:** 2017-06-28

**Authors:** Jeffrey F. Bromaghin, Suzanne M. Budge, Gregory W. Thiemann, Karyn D. Rode

**Affiliations:** ^1^ Alaska Science Center U.S. Geological Survey Anchorage AK USA; ^2^ Process Engineering and Applied Science Dalhousie University Halifax NS Canada; ^3^ Faculty of Environmental Studies York University Toronto ON Canada

**Keywords:** diet estimation, food web, QFASA, qfasar, quantitative fatty acid signature analysis

## Abstract

Knowledge of animal diets provides essential insights into their life history and ecology, although diet estimation is challenging and remains an active area of research. Quantitative fatty acid signature analysis (QFASA) has become a popular method of estimating diet composition, especially for marine species. A primary assumption of QFASA is that constants called calibration coefficients, which account for the differential metabolism of individual fatty acids, are known. In practice, however, calibration coefficients are not known, but rather have been estimated in feeding trials with captive animals of a limited number of model species. The impossibility of verifying the accuracy of feeding trial derived calibration coefficients to estimate the diets of wild animals is a foundational problem with QFASA that has generated considerable criticism. We present a new model that allows simultaneous estimation of diet composition and calibration coefficients based only on fatty acid signature samples from wild predators and potential prey. Our model performed almost flawlessly in four tests with constructed examples, estimating both diet proportions and calibration coefficients with essentially no error. We also applied the model to data from Chukchi Sea polar bears, obtaining diet estimates that were more diverse than estimates conditioned on feeding trial calibration coefficients. Our model avoids bias in diet estimates caused by conditioning on inaccurate calibration coefficients, invalidates the primary criticism of QFASA, eliminates the need to conduct feeding trials solely for diet estimation, and consequently expands the utility of fatty acid data to investigate aspects of ecology linked to animal diets.

## 1 INTRODUCTION

Estimation of diet composition (hereafter diet) is a critically important topic, as knowledge of animal diets informs numerous aspects of their ecology on scales ranging from individuals to communities, and consequently remains an active area of research in quantitative ecology. Several methods of diet estimation are practiced by ecologists, with the examination of scats or digestive tracts (e.g., Bowles, Schulte, Tollit, Deagle, & Trites, 2011; Marshall et al., 2010; Roberts & Lalas, 2015) and models based on biochemical data such as stable isotopes and fatty acids (e.g., Brett, Eisenlord, & Galloway, 2016; Haynes et al., 2015) being especially common. Such a diverse collection of methods has likely developed because no one method is ideal for all circumstances, but rather each has inherent strengths or limitations that affect its suitability for a particular investigation (e.g., Bowen & Iverson, 2013; Klare, Kamler, & Macdonald, 2011; Phillips et al., 2014).

Quantitative fatty acid signature analysis (QFASA; Iverson, Field, Bowen, & Blanchard, 2004) has become a popular method of diet estimation, particularly for marine species (Bowen & Iverson, 2013). The fundamental unit of information in QFASA is a vector of proportions summarizing the fatty acid composition of lipids, termed a signature. Predator signatures are modeled as mixtures of the signatures of potential prey, and diet is estimated as the prey mixture that minimizes a measure of distance between the observed and modeled predator signatures. Constants called calibration coefficients are used to adjust for the differential metabolism of individual fatty acids.

Quantitative fatty acid signature analysis has several characteristics that partially distinguish it from other methods and may affect its suitability for a particular investigation. The method produces quantitative estimates of diet composition, with associated measures of precision, and the estimates are pertinent to a longer period of time than estimates obtained using many other methods (Budge, Iverson, & Koopman, 2006). Sampling can be nonlethal and requires only the collection of a relatively small portion of fat tissue, although lipid stratification and the region of the body sampled can be important with some species (e.g., Guerrero et al., 2016; Lambert, Meynier, Donaldson, Roe, & Morel, 2013; Mohan, Mohan, Connelly, Walther, & McClelland, 2016). Diet estimation usually involves a relatively large number of fatty acids, which allows the contribution of a corresponding number of prey types to be estimated and greatly reduces the problem of underdetermined systems common with stable isotope models (Brett, 2014; Phillips & Gregg, 2003). The model is based on two key assumptions: (1) representative signatures of all potential prey types are available, and (2) the calibration coefficients are known. Computer simulations have confirmed that the model performs well when the assumptions are met (Bromaghin, Budge, Thiemann, & Rode, 2016), although analytical choices can influence estimates and there are only general guidelines for which choices might be preferred (Bromaghin, Budge, & Thiemann, 2016; Bromaghin, Rode, Budge, & Thiemann, 2015). However, feeding trials with captive animals have been necessary to estimate the calibration coefficients, and their accuracy to estimate the diets of wild predators cannot be verified and must be explicitly assumed (Bromaghin, Budge, Thiemann, & Rode, 2016). Finally, for investigation of predators that might consume numerous prey types, a significant investment may be required to develop an adequate collection of prey signature data, termed a library.

The unverifiable assumption that calibration coefficients are known has received considerable attention in the literature. Several investigators have found diet estimates to be sensitive to the choice of calibration coefficients (e.g., Budge, Penney, & Lall, 2012; Haynes et al., 2015; Meynier, Morel, Chilvers, Mackenzie, & Duignan, 2010; Nordstrom, Wilson, Iverson, & Tollit, 2008; Wang, Hollmén, & Iverson, 2010), and simulation studies have demonstrated that errors in their values can bias diet estimation (Bromaghin, Budge, Thiemann, & Rode, 2016). In feeding trials, calibration coefficient estimates have been found to vary by the species of the consumer and various aspects of feeding trial design (e.g., Budge, Penny, & Lall, 2011; Rosen & Tollit, 2012; Thiemann, Iverson, & Stirling, 2008; Wang et al., 2010). Further, fatty acids may be deposited or turnover at different rates in different tissues (Mohan et al., 2016; Nordstrom et al., 2008), and diet composition, physiological state, and the age of an animal can affect fatty acid metabolism (Williams & Buck, 2010). Designing a feeding trial to develop calibration coefficients that are robust to so many factors is complex and perhaps not even feasible. Such difficulties with calibration coefficients have caused some investigators to question the utility of QFASA (e.g., Happel et al., 2016; Rosen & Tollit, 2012; Williams & Buck, 2010).

**Figure 1:**
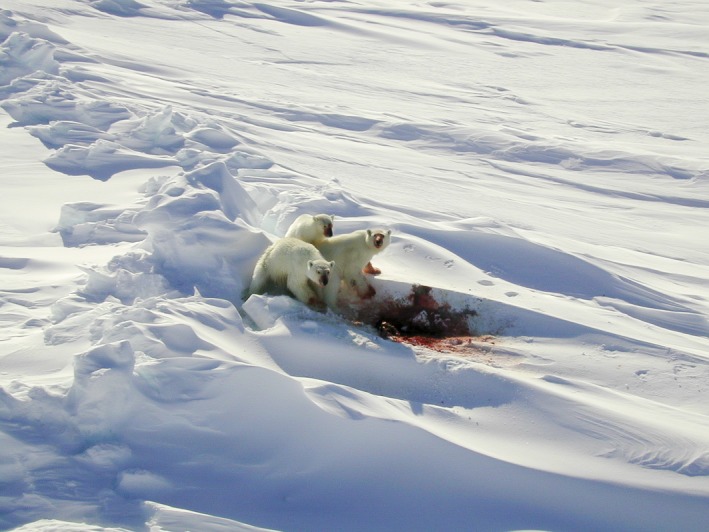
A polar bear (*Ursus maritimus*) family feeding on a ringed seal (*Phoca hispida*). Photograph credit: U.S. Geological Survey, Alaska Science Center. Previously published by Ecology and Evolution 5:1249–1262

We present a new model that allows simultaneous estimation of both diets and calibration coefficients based only on fatty acid signature samples from predators and potential prey. The primary requirements are the availability of a suitable prey library and a predator sample that exceeds a minimum number of animals, which depends upon the number of prey types and fatty acids used. The performance of our model was explored using constructed test cases, in which the true values of diet proportions and calibration coefficients were known, based on two prey libraries previously used to investigate the performance properties of QFASA diet estimators (e.g., Bromaghin, Budge, Thiemann, & Rode, 2016). Diet composition and calibration coefficients were also estimated for a sample of Chukchi Sea polar bears (Figure. 1; *Ursus maritimus*) whose diets were previously estimated (Rode et al., 2014) using calibration coefficients derived from a mink (*Neovison vison*) feeding trial (Thiemann et al., 2008).

## 2 MATERIALS AND METHODS

### 2.1 The model

Our notation is a minor extension of that of Iverson et al. (2004). Let ${\overset{¯}{x}}_{ik}$ = the proportion for fatty acid k in the mean signature of prey type *i*;* i* = 1, 2, …, *I*;* k* = 1, 2, …, *K*;* y*_*jk*_ = the proportion for fatty acid *k* in the signature of predator *j*;* j* = 1, 2, …, *J*;* c*_*k*_ = the calibration coefficient for fatty acid k, common to all predators; and π_*ji*_ = the proportion of prey type *i* in the diet of predator *j*.

**Figure 2:**
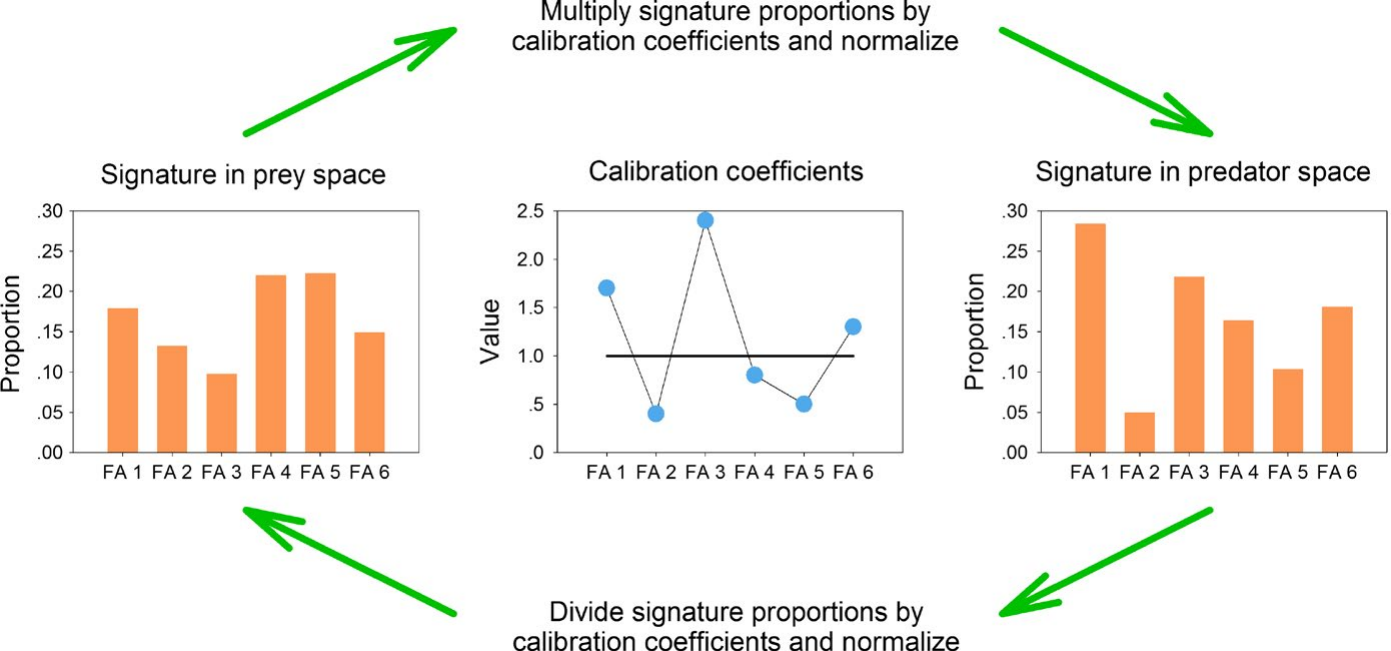
An example with six fatty acids (FA) illustrating how calibration coefficients are used to transform signatures between the predator and prey spaces

Calibration coefficients are used to adjust signature proportions for the effects of fatty acid metabolism, providing a one‐to‐one mapping between the prey and predator spaces (Figure. 2). Diet estimation can occur in either space because metabolic effects have been accounted for and the signatures made comparable, although estimates obtained in the two spaces may differ (Bromaghin, Rode, Budge, & Thiemann, 2015). For example, Iverson et al. (2004) divided predator signatures by the calibration coefficients to transform the signatures to the prey space, while Bromaghin et al. (2013) multiplied prey signatures by the calibration coefficients to transform the signatures to the predator space.

We performed estimation in the predator space, so calibration coefficients (*c*_*k*_) were used to transform mean prey signatures (${\overset{¯}{x}}_{ik}$) to the predator space,

$${\overset{¯}{x}}_{ik}^{t}\;=\;\frac{c_{k}{\overset{¯}{x}}_{ik}}{\sum_{m}c_{m}{\overset{¯}{x}}_{im}}.$$

Predator signatures were modeled as a mixture of the transformed prey signatures (${\overset{¯}{x}}_{ik}^{t}$), with diet proportions (π_*ji*_) as the weights,

$${\hat{y}}_{jk}\;=\;\sum_{i}\pi_{ji}{\overset{¯}{x}}_{ik}^{t},$$

and diet proportions and calibration coefficients were estimated by minimizing the Aitchison distance (Aitchison, 1986) between the observed and modeled signatures summed over all predators.

$$Q\;=\;\sum_{j}\sqrt{\sum_{k}{\left(\text{log}\left[\frac{{\hat{y}}_{jk}}{\text{gm}({\hat{y}}_{j})}\right]\;-\;\text{log}\left[\frac{y_{jk}}{\text{gm}(y_{j})}\right]\right)}^{2},}$$

where gm(s) is the geometric mean of the K fatty acid proportions in signature s. The key differences between this model and prior QFASA models are that the calibration coefficients are unknown parameters to be estimated, rather than known constants, and the distance between observed and modeled signatures is summed over all predators in the sample.

There are J*(*K*−1) degrees of freedom in the predator signature data, losing one degree of freedom for each predator because the proportions in each signature must sum to 1. The diet proportions for each predator must also sum to 1, so there are J*(*I*−1) unknown diet proportions. Only the relative magnitudes of the calibration coefficients are informative for diet estimation, that is, multiplying their values by any constant produces an identical mapping between the prey and predator spaces, so one identifiability constraint must be placed on them and there are therefore *K*−1 free calibration coefficients. The J*(*I*−1) diet proportions and *K*−1 calibration coefficients comprise the parameters of the model. Conceptually, all parameters are estimable if the degrees of freedom equal or exceed the number of parameters, which can be expressed as *J* ≥ (*K*−1)/(*K*−*I*), for *K* > *I*. The number of prey types will always exceed 1, so this minimum threshold exceeds 1 and will increase as the difference between the number of fatty acids and the number of prey types decreases.

All data processing was performed using MATLAB (version 2016b, www.mathworks.com/), and the objective function Q was minimized using TOMLAB SNOPT software (version 8.0, www.tomopt.com/tomlab/). Initial values for all diet proportions were set to the inverse of the number of prey types, 1/*I*, and initial values for all calibration coefficients were set to 1. For the identifiability constraint on the calibration coefficients, we arbitrarily chose to constrain their sum to equal the number of fatty acids, K. Calibration coefficients were additionally constrained to be at least 0.02 to bound them from zero and avoid potential computational problems during parameter estimation. Finally, the diet proportions of each predator were constrained to be non‐negative and sum to 1.

### 2.2 Prey libraries

Our analyses were based on two prey libraries with quite different characteristics that have previously been used to investigate the performance of QFASA diet estimators (e.g., Bromaghin, Budge, Thiemann, & Rode, 2016). The marine mammal (hereafter mammal) library (Bromaghin et al., 2015a,b) was comprised of 357 signatures from seven species that have been used to estimate the diets of Chukchi Sea polar bears (Rode et al., 2014). Several prey types in this library have reasonably distinct signatures, although there is some confounding between the ice seal species, especially ribbon seal *Histriophoca fasciata* and spotted seal *Phoca vitulina* (Bromaghin, 2017). For the mammal library, we used the 31 fatty acids previously used by Thiemann et al. (2008) to estimate polar bear diets. The second library was the Scotian Shelf fish and shellfish (hereafter fish) library, comprised of 954 signatures from 28 species (Bromaghin et al., 2015b; Budge, Iverson, Bowen, & Ackman, 2002). The fish library is considerably more complex because of the larger number of prey types and the confounding that exists among the signatures of several prey types (Bromaghin, Rode, et al., 2015). With the fish library, we used the extended dietary suite of 41 fatty acids (Iverson et al., 2004), which is nearly identical to the suite of 39 fatty acids that have been used with expanded versions of this library (e.g., Beck, Iverson, Bowen, & Blanchard, 2007).

With both libraries, fatty acid proportions that were missing or equal to zero were replaced by a small constant (0.005), a common strategy in QFASA because distance measures for compositional data often involve logarithms and so are not defined if any proportions equal zero. The sum of the proportions for the fatty acids used in diet estimation was computed for each signature, and each signature was then augmented with an additional proportion equal to one minus that sums so that the proportions in each augmented signature summed to one, which has been found to reduce bias in some circumstances (Bromaghin, Budge, Thiemann, 2016). Consequently, signatures were comprised of 32 and 42 proportions with the mammal and fish libraries, respectively. We therefore needed a minimum of *J* = 2 predators for the mammal library and *J* = 3 predators for the fish library for all parameters to be estimable.

### 2.3 Example diets

**Figure 3:**
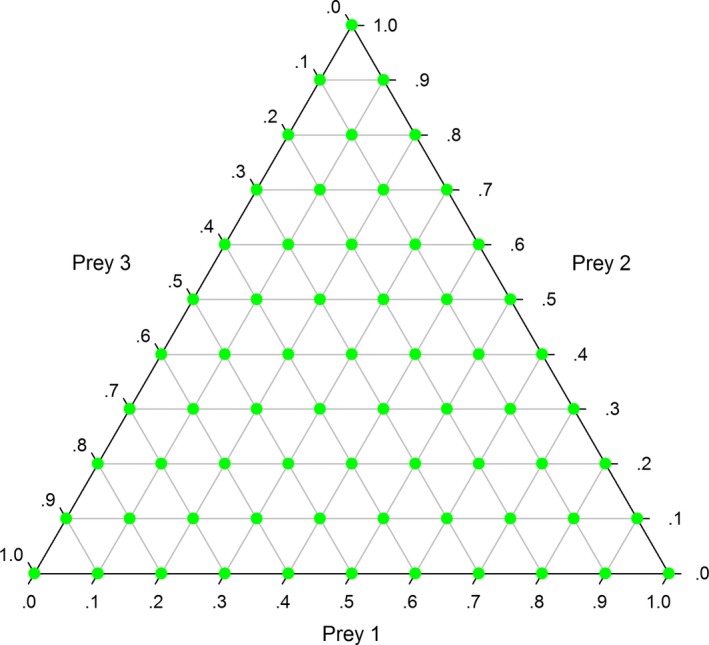
A ternary plot illustrating a grid of diet proportions regularly spaced throughout the range of all possible diets comprised of up to three prey types, with an increment of 0.1 between proportions. Similar diet grids with the larger mammal and fish prey libraries were used to establish example diets to test the performance of the model

We expected the model to perform optimally when the number of predator signatures was well above the minimum sample size threshold and predator diets were highly diverse. Consequently, we established a large set of diverse predator diets for each library by selecting a grid of diets regularly spaced throughout the range of all diets possible with each library (Bromaghin, Budge, Thiemann, 2016). As an example, a diet grid for three prey types having diet proportions equally spaced by an increment of 0.10 is illustrated in Figure. 3. The diet grids used in our analyses were generated using the make_diet_grid function of the R package qfasar (version 1.1.0, Bromaghin, 2017) with a diet increment of 0.25, which resulted in grids of 210 and 31,465 diets with the mammal and fish libraries, respectively. We randomly selected a subset of 210 diets from the fish library grid to reduce the number of diets to a manageable number. For each library and its suite of fatty acids, calibration coefficients were established by drawing a random sample from a chi‐square density with one degree of freedom and scaling them to sum to the number of fatty acids used with each library. Each of the example diets was then used to compute a predator signature in the prey space as a mixture of the mean prey signatures weighted by the diet proportions (Iverson et al., 2004), and the calibration coefficients were used to transform each signature from the prey space to the predator space. The resulting predator signatures and the mean prey signatures were then used as data inputs to the new model described above, and diet proportions and calibration coefficients were simultaneously estimated. There were a total of 1,291 and 5,711 diet proportion and calibration coefficient parameters in the models based on the mammal and fish libraries, respectively.

Test cases that we expected to be more challenging for the model were based on the realistic diets of adult female and male polar bears (mammal library) and spring‐ and fall‐sampled, female and male gray seals (*Halichoerus grypus*; fish library) used as test cases by Bromaghin, Rode et al. (2015). Estimated diets for subadult female and male polar bears (Rode et al., 2014) were added so that we had four realistic diets for each library. These test cases were expected to be more difficult because the number of diets (four) was much closer to the minimum sample size threshold of each library (either two or three), and the diets were considerably less diverse than the gridded diet test cases. Using the four realistic diets for each library, a process identical to that previously described for the gridded diets was used to generate predator signatures in the prey space, map them to the predator space using the same calibration coefficients, and estimate both diets and calibration coefficients. There were 55 and 149 parameters in the models based on the mammal and fish libraries, respectively.

For both gridded and realistic diet test cases, the true values of the diet proportions and calibration coefficients were known. Given the large number of diet proportions in the diet grid analyses, we computed differences between the estimated and true proportions (error or statistical bias) and graphically summarized their distribution. Because of the smaller number of diet proportions in the analyses based on realistic diets, we graphically compared the true and estimated proportions for each prey component of the four diets. In both cases, we graphically compared estimated calibration coefficients with the true values.

### 2.4 Chukchi Sea polar bears

We estimated the diets and calibration coefficients for a sample of 154 polar bears from the Chukchi Sea (Regehr, Wilson, Martin, & Rode, 2016) using the mammal library. The diets of these bears were previously estimated (Rode et al., 2014) using the original QFASA model, the mammal library, and calibration coefficients derived from a mink feeding trial (Thiemann et al., 2008). The bear signatures were prepared for analysis using the same methods of zero replacement and signature augmentation previously described for the prey libraries. Individual bear diets and calibration coefficients were estimated using the new model, and the mean diet was computed from the individual estimates for each of four age–sex classes: adult females, adult males, subadult females, and subadult males. In this case, the true diets and calibration coefficients were unknown. Consequently, we compared our estimated calibration coefficients with the values derived from the mink feeding trial (Thiemann et al., 2008), scaled to a common sum to make the comparison meaningful. Our estimates of diet composition were compared to a second set of estimates also obtained using our new model, but with the calibration coefficients constrained to equal the marine‐fed values derived from the mink feeding trial.

## 3 RESULTS

**Figure 4:**
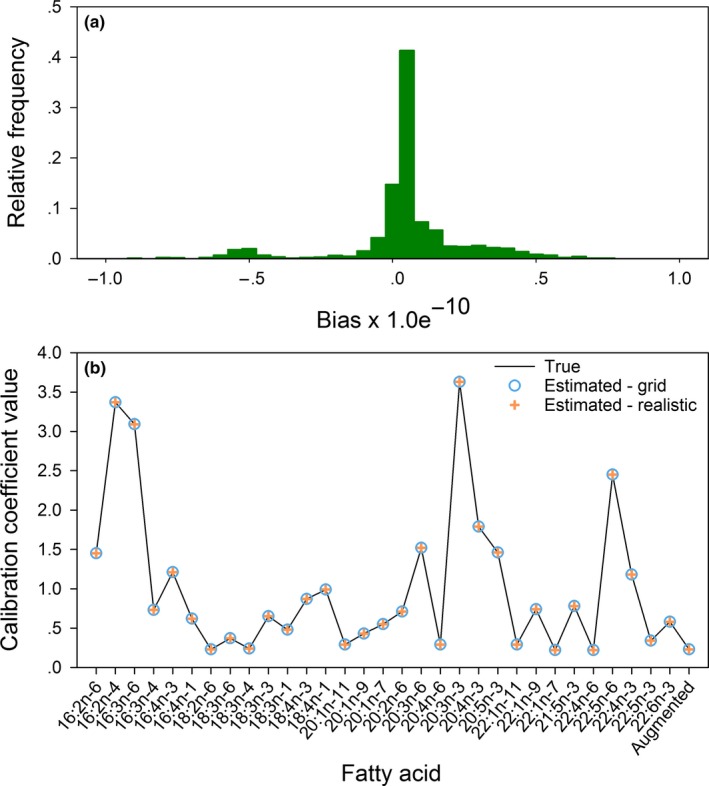
Estimation results for the grid of 210 diets based on the mammal library: (a) the distribution of bias among the estimated diet proportions, and (b) true values of the calibration coefficients used to construct predator signatures based on the mammal library, with estimates obtained in the diet grid and realistic diet analyses

**Figure 5:**
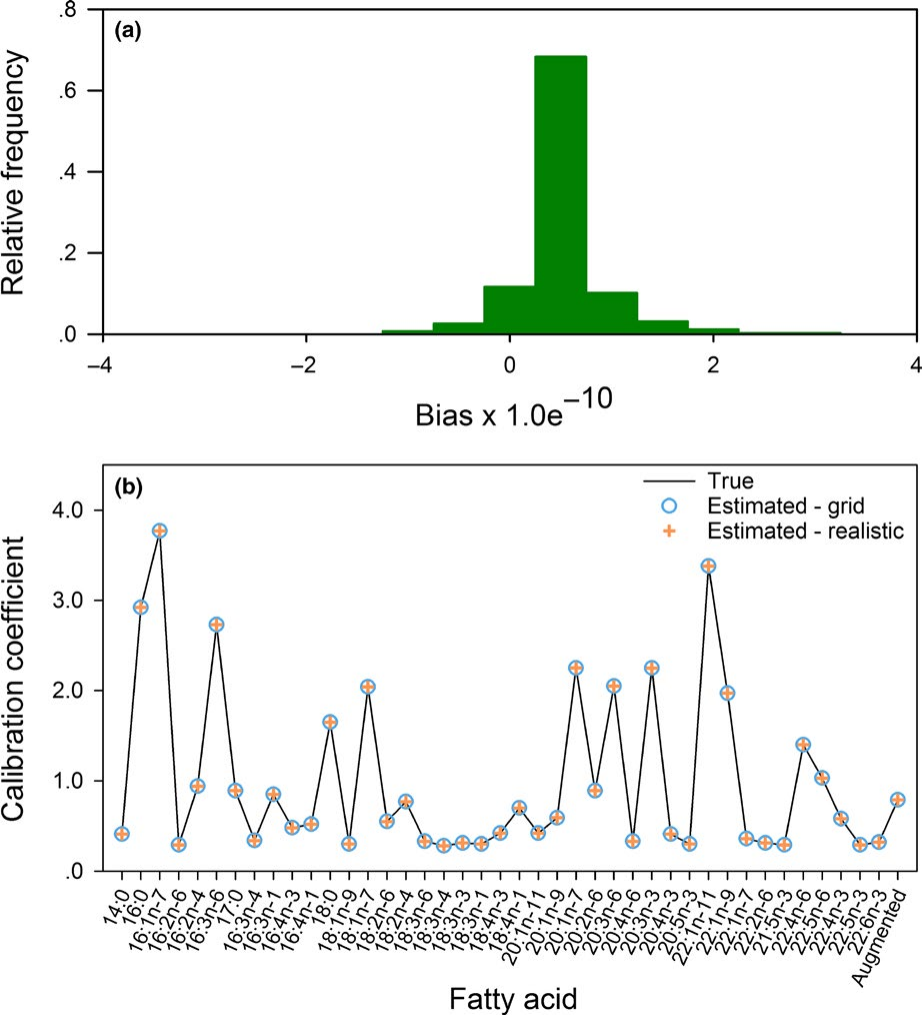
Estimation results for the grid of 210 diets based on the fish library: (a) the distribution of bias among the estimated diet proportions, and (b) true values of the calibration coefficients used to construct predator signatures based on the mammal library, with estimates obtained in the diet grid and realistic diet analyses

In the diet grid analysis with the mammal library, the minimized value of the objective function was effectively zero (<1.0e^−18^), so the model came very close to fitting the predator signature data perfectly. The estimated diet proportions and calibration coefficients (Figure. 4) were effectively unbiased, with errors on the order of 1.0e^−10^. The results obtained with the fish library were similarly accurate. The value of the minimized objective function was <1.0e^−18^, and the diet proportions and calibration coefficients (Figure. 5) were estimated with essentially no error.

**Figure 6:**
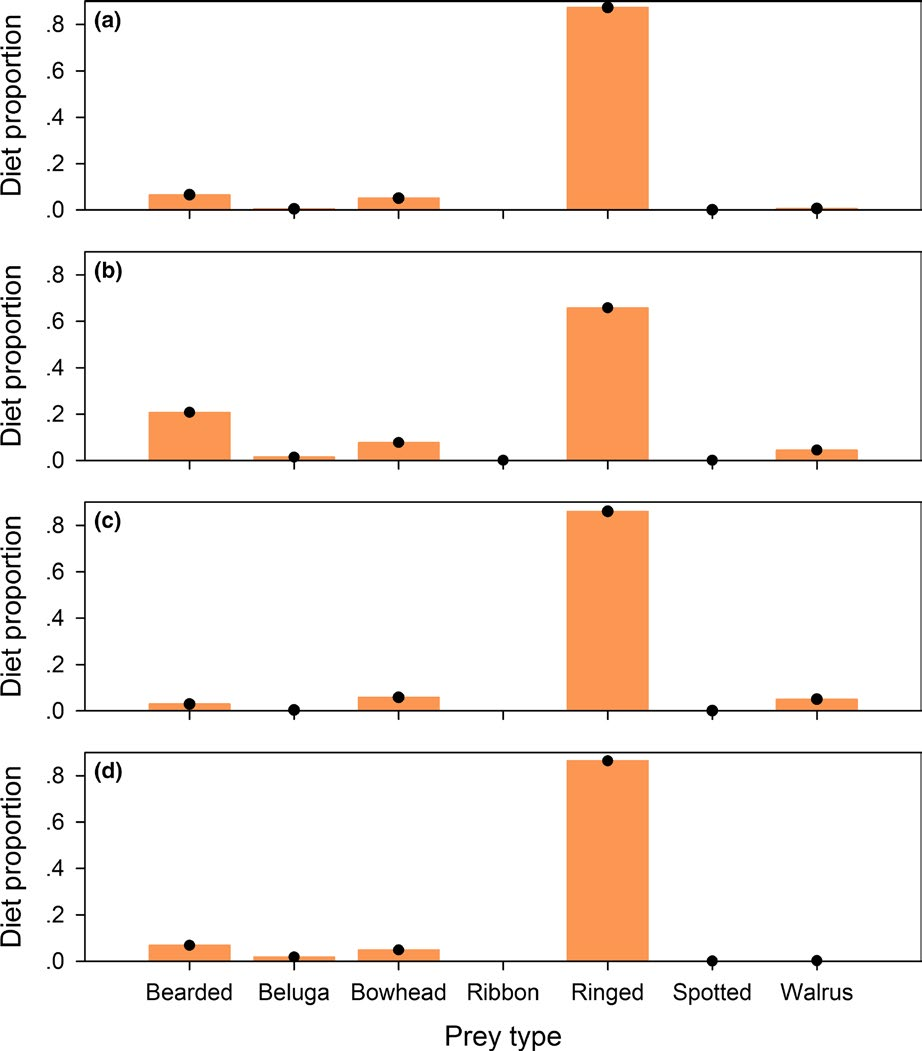
True (bars) and estimated (circles) diet proportions for the realistic Chukchi Sea polar bear diet analysis with the mammal library, for (a) adult females, (b) adult males, (c) subadult females, and (d) subadult males

**Figure 7:**
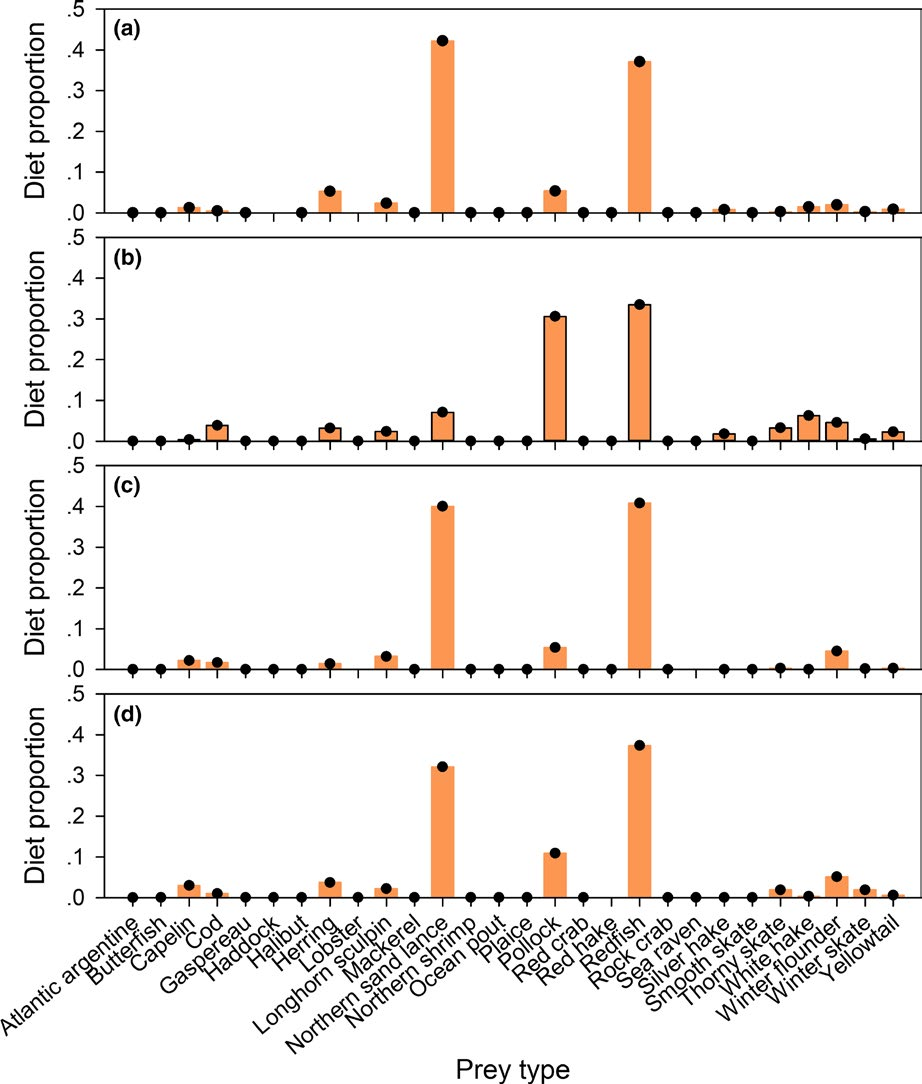
True (bars) and estimated (circles) diet proportions for the realistic gray seal diet analysis with the fish library, for (a) spring‐sampled females, (b) spring‐sampled males, (c) fall‐sampled females, and (d) fall‐sampled males

Estimates for the realistic diets also had very little error. With the mammal library, the value of the minimized objective function was <1.0e^−18^, and estimation errors for the calibration coefficients (Figure. 4b) and diet proportions (Figure. 6) were on the order of 1.0e^−10^. With the fish library, the minimized objective function was slightly greater than zero (2.3 × 10^−12^) and the optimization routine returned a warning that an improved solution could not be found, which jointly implied that a good solution was found but the termination criteria were not fully satisfied. Errors in the estimated diet proportions were slightly larger than in the other cases, but still inconsequential from a practical perspective, ranging from −3.5e^−5^ to 4.1e^−5^ (Figure. 7), and the calibration coefficient estimates had errors of a similar magnitude (Figure. 5b).

**Figure 8:**
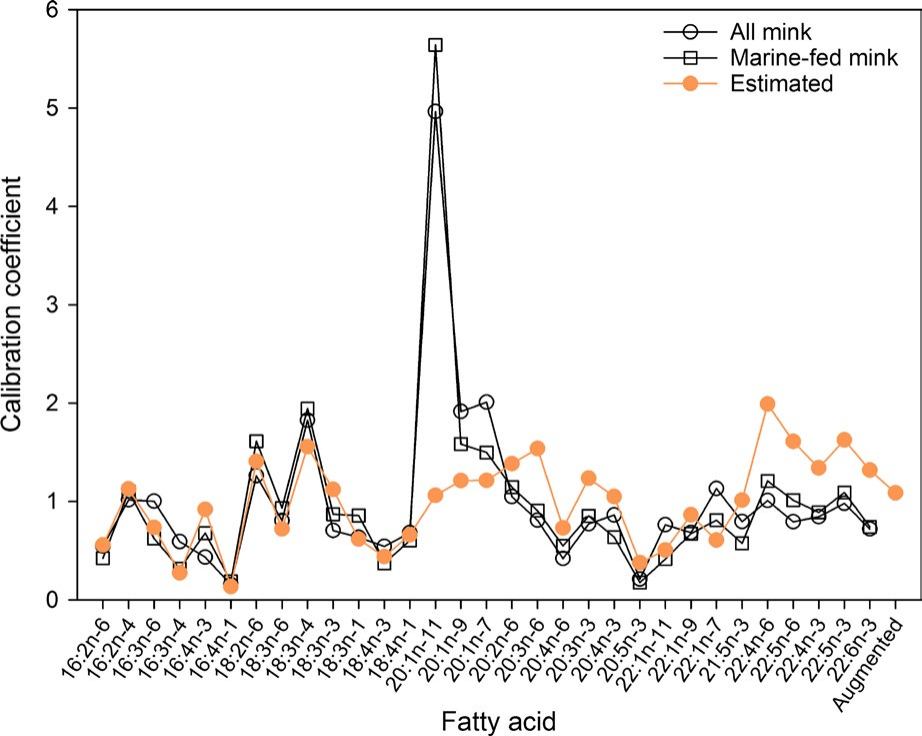
Unconditional estimates of calibration coefficients for Chukchi Sea polar bears, along with the two sets of values derived from a mink feeding trial. The feeding trial values have been scaled so they sum to the number of fatty acids to allow a meaningful comparison

**Figure 9:**
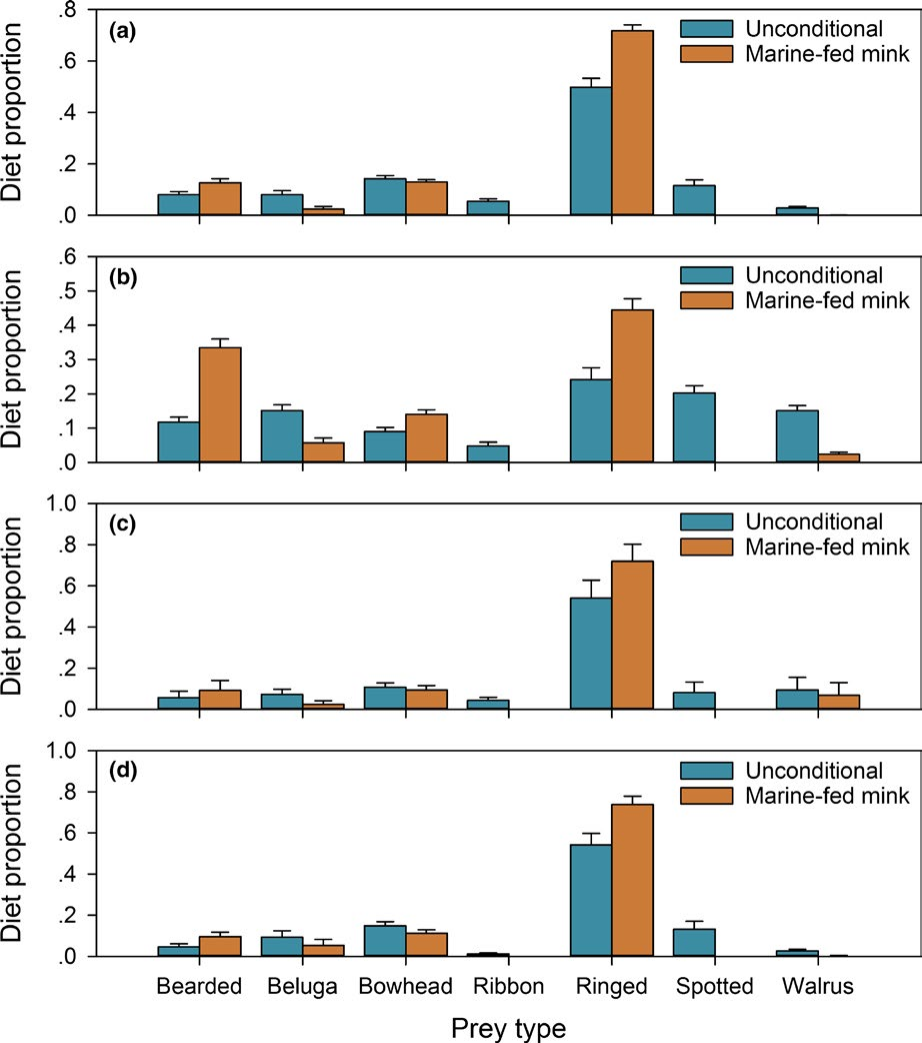
Mean estimated diet of Chukchi Sea polar bears obtained with unconditional estimation and by conditioning on the marine‐fed mink calibration coefficients for (a) adult females, (b) adult males, (c) subadult females, and (d) subadult males. Error bars represent one standard error of the mean

With the Chukchi Sea polar bear data and the marine mammal library, the estimated calibration coefficients for many fatty acids were somewhat similar to the values derived from the mink feeding trial (Thiemann et al., 2008; Figure. 8). The most notable exception was fatty acid 20:1n‐11, for which our estimated calibration coefficient was substantially smaller than either of the feeding trial estimates. In addition, our estimates for the 22‐carbon polyunsaturated fatty acids tended to be somewhat larger than the corresponding feeding trial estimates. Our unconditional diet estimates were more diverse than estimates conditioned on the marine‐fed mink calibration coefficients (Figure. 9) and tended to have larger contributions from beluga whale (*Delphinapterus leucas*), ribbon seal, spotted seal, and walrus (*Odobenus rosmarus*), smaller contributions from bearded seal (*Erignathus barbatus*) and ringed seal (*Pusa hispida*), and similar contributions from bowhead whale (*Balaena mysticetus*). Our second set of estimates conditioned on the marine‐fed mink calibration coefficients were close to previous estimates obtained using similar methods (Bromaghin, Rode et al., 2015).

## 4 DISCUSSION

Our analyses of example diets, constructed under known conditions with two prey libraries having substantially different characteristics, demonstrate that simultaneous estimation of diet composition and calibration coefficients based only on signature samples from wild predator and prey is not only feasible, but also highly accurate. In all four cases in which the true values of the diets and calibration coefficients were known, both sets of parameters were estimated with essentially no error.

The key feature of our new model is the simultaneous estimation of multiple predator diets and a set of calibration coefficients common to all predators. In a sense, predator signature data contain more information about calibration coefficients than diets, because each signature contains information about the calibration coefficients, but only about the diet of an individual predator. For example, a fatty acid with a relatively small or large calibration coefficient creates a strong signal in predator signatures by either decreasing or increasing the prevalence of that fatty acid, and that signal is created irrespective of the prey consumed. Consequently, the influences of calibration coefficients and diets are separately identifiable if a sufficient number of predators are considered simultaneously. Separate identifiability is not possible with prior QFASA models because each predator diet is estimated independently. In a single predator model, diet proportions and calibration coefficients are completely confounded, always occurring together as either a product (predator space estimation) or ratio (prey space estimation), so only the product or ratio can be estimated. Single predator models must therefore condition on specific values for the calibration coefficients in order for diet proportions to be estimable. As an aside, we note that analysis of feeding trial data essentially inverts that process, conditioning on a known diet to estimate calibration coefficients.

In our analyses based on realistic diets, all parameters were successfully estimated with the signatures of only four predators. However, in the analysis with the fish library, the warning received from the optimization routine, a minimized objective function somewhat greater than zero, and slightly greater, though inconsequential, bias likely indicated that the model was challenged in that case. The sample of four predators was only marginally greater than the theoretical minimum of three predators for the fish library, which may have caused some difficulty. In practice, having a larger number of predators is recommended and doing so can be expected to increase estimation accuracy, because each predator contributes some information about the calibration coefficients, as described above. It is also important to realize that some diversity in the predator data is necessary for both diet proportions and calibration coefficients to be estimable. For example, in an extreme case in which all predator signatures were identical, the effective sample size would be one, diet proportions and calibration coefficients would be completely confounded as in a single predator model, and estimation would fail. For that reason, it is probably best to think of the minimum sample size threshold as applying to the number of distinct diets, rather than the number of predators, contained within the sample, although data from wild predators seem unlikely to be sufficiently homogeneous for this distinction to be pertinent. When working with a small number of predators, especially if their signatures are quite similar, it would be prudent to verify that starting with multiple, diverse guesses of the parameter values converge to a common estimate.

The ability to estimate diets without calibration coefficients derived from a feeding trial is a major breakthrough with profound benefits. Conducting a feeding trial requires a facility with the capability to properly house and care for an adequate number of animals. Feeding trials must be conducted over lengthy periods of time, as the relationship between consumer diet and lipid reserves takes time to develop and stabilize (e.g., Budge et al., 2012). Consequently, feeding trials are often time‐consuming and expensive. In addition, a number of animal welfare concerns could arise from holding animals, feeding a controlled diet, or sample acquisition. Such issues can preclude working with rare species held in zoos and similar facilities that prioritize animal welfare, even though data from such species might be extremely valuable to support field investigations.

Although our model avoids the need to conduct a feeding trial to estimate calibration coefficients, the prey library remains a critically important data input. For accurate estimation of diets, the prey library must contain representatives of all prey potentially consumed by predators. To the degree possible, prey types should be defined to minimize differences among signatures within prey types and maximize differences between prey types. In addition, our model assumes that the predators share a common set of calibration coefficients. If there is reason to suspect that calibration coefficients differ by sex, age, or similar factors, predator data can be partitioned into subsamples, and estimation can be performed separately for each.

The similarity between our estimates of calibration coefficients for the Chukchi Sea polar bears and the estimates derived from the mink feeding trial (Thiemann et al., 2008) may provide some assurance that both sets of estimates reflect related metabolic processes. The greatest difference between the calibration coefficients occurred with fatty acid 20:1n‐11, for which our estimate was substantially smaller than either of the feeding trial estimates, although other less striking differences were also found. The cause of these differences is unknown, but likely originates from some aspect of the feeding trial design, such as characteristics of the diets fed, or differences in the physiology of mink and polar bears. In a prior analysis of the polar bear data, Bromaghin, Rode et al. (2015) reported that many polar bear fatty acid proportions were outside the range of the proportions in the transformed prey library. This finding is conceptually impossible for the QFASA mixing model if assumptions are met, and strongly suggests that the mink‐derived calibration coefficients are not wholly suitable for Chukchi Sea polar bears.

Our unconditional estimates of Chukchi Sea polar bear diets were more diverse than estimates conditioned on the marine‐fed mink calibration coefficients, with more ribbon seal, spotted seal, and walrus and less bearded seal and ringed seal than previously reported (Rode et al., 2014). Ribbon seal, spotted seal, and walrus all occur in the Chukchi Sea during at least some portion of the year (Simpkins, Hiruki‐Raring, Sheffield, Grebmeier, & Bengtson, 2003), but their availability to polar bears has been thought to be limited by their use of land haul‐outs and selection of sea ice habitats that are less preferred by polar bears (Lowry, Frost, Davis, DeMaster, & Suydam, 1998; Simpkins et al., 2003; Wilson, Regehr, Rode, & Martin, 2016). Unpublished field observations of polar bear kill sites during the spring (March to May) confirm the importance of bearded and ringed seals as prey, as well as the rare use of walrus and beluga whale, but have not documented consumption of ribbon or spotted seal. However, polar bear fat biopsies collected in spring are thought to reflect prey consumption over a period of months (Budge et al., 2006), perhaps extending as far back as early winter or autumn when other species may be more available than in the spring (Simpkins et al., 2003). For example, polar bears have been observed attacking walruses hauled out on the coast in the fall, and Alaskan hunters have reported the consumption of spotted seals in winter (Voorhees, Sparks, Huntington, & Rode, 2014). The changing phenology of Arctic sea ice (Serreze, Crawford, Stroeve, Barrett, & Woodgate, 2016) is altering polar bear's behavior and habitat selection (Rode et al., 2015; Ware et al., 2017) and, combined with the ecosystem response to ice loss (e.g., Feng, Ji, Campbell, Ashjian, & Zhang, 2016; Moore, 2016), may be shifting the composition of prey species available to polar bears (e.g., Beatty et al., 2016; Galicia, Thiemann, Dyck, Ferguson, & Higdon, 2016).

## 5 CONCLUSIONS

We have conclusively demonstrated that predator diet composition and calibration coefficients can be simultaneously estimated based only on signature samples from wild predators and their potential prey. This methodological breakthrough has profound implications for this discipline of quantitative ecology, eliminating bias in diet estimation caused by conditioning on calibration coefficients that may be inaccurate, nullifying the criticism of QFASA that has been most prevalent in the literature, and substantially increasing the utility of fatty acid data to investigate aspects of predator ecology linked to their diets. Although feeding trials will undoubtedly continue to provide useful insights into animal physiology, they are not required for diet estimation. Our modeling approach is easily adaptable for use with models based on other data types, so long as the models are not underdetermined.

## AUTHOR CONTRIBUTIONS

JFB developed the model, performed the analyses, and led manuscript preparation. SMB, GWT, and KDR provided data and assisted with the interpretation of results and manuscript preparation.

## CONFLICT OF INTEREST

None declared.
